# Introduction of a standardized semi-quantitative body condition scoring system for cattle and pigs in the Democratic Republic of the Congo

**DOI:** 10.1016/j.vas.2026.100601

**Published:** 2026-02-17

**Authors:** Paige Rudin Kinzie, Sydney Merritt, Boniface Lombe, Megan Halbrook, Danae Witte, Emmanuel Hasivirwe Vakaniaki, Saraina Adam, Alex Madidi L’k’ye, Patrick K. Mukadi, Milette Mfwankang, Jean Mona, Daddy Kanonge, Carleigh Melofchik, Jean-Jacques Muyembe-Tamfum, Nicole A. Hoff, Lisa E. Hensley, Placide Mbala-Kingebeni, Augustin Twabela, Anne W. Rimoin

**Affiliations:** a Purdue University, College of Veterinary Medicine, West Lafayette, Indiana, USA; b University of Minnesota, School of Public Health, Minneapolis, Minnesota, USA; c Department of Epidemiology, Jonathan and Karin Fielding School of Public Health, University of California, Los Angeles, California, USA; d Laboratoire Vétérinaire Central de Kinshasa, Kinshasa, Democratic Republic of the Congo; e Faculté de Médecine Vétérinaire, Université Pédagogique Nationale, Kinshasa, Democratic Republic of the Congo; f College of Veterinary Medicine, University of Florida, Gainesville, Florida, USA; g Institut National de Recherche Biomédicale, Kinshasa, Democratic Republic of the Congo; h Zoonotic and Emerging Disease Research Unit, National Bio and Agro-Defense Facility, USDA Agricultural Research Service (ARS), Manhattan, Kansas, USA; i Microbiology Service, Department of Medical Biology, Cliniques Universitaires de Kinshasa, University of Kinshasa, Democratic Republic of the Congo

**Keywords:** Livestock Health, Emerging zoonoses, Public Health, Surveillance, One Health

## Abstract

To improve the standardization of veterinary assessments of livestock health and welfare across the Democratic Republic of the Congo (DRC), we piloted and validated the implementation of a multispecies semiquantitative six-point body condition scoring (BCS) system for use in farms, abattoirs, and free-range village herds. BCS systems indicating animal energy balance and overall systemic health are critical to rapid and accurate veterinary surveillance to prevent the spread of zoonotic disease and ensure a safe food supply, among other One Health implications. Livestock species may be intermingled in Congolese agricultural settings, requiring a standard BCS scale; we harmonized existing species-specific scales to improve traditional approaches limited to qualitative assessment only. With a team of Congolese veterinarians and technicians, we completed 124 site visits across nine provinces between June 2023 and July 2024, comparing BCS ratings with various animal health indicators to identify patterns and relationships across 1038 swine and 1157 bovine subjects. Results indicate BCS ratings varied significantly by site type (p < 0.001) and age (p < 0.001), as well as between animals with reported histories of illness (p < 0.001) and lesions at the time of sampling (p = 0.089). At selected sites, the investigation team achieved an agreement with a maximum difference of 1 score unit between raters in an assessment of inter-rater reliability (n = 36, κ = 0.47, 95 % CI: 0.22–0.73) and qualitatively reported improved assessment precision with the six-point system compared to previous unstandardized processes.

## Introduction

Body condition scoring (BCS) is a widely accepted subjective, semi-quantitative, and standardized tool to rapidly assess an animal’s physical condition to gain insights about its health, nutrition, and welfare status. Typically a morphometric assessment of adiposity, a BCS standard is developed for a specific species and, often, specific breeds, designed to accurately represent individuals in a majority of the population with a handful of discrete ratings (e.g., 1–5 or 1–9 for domestic small animal species) (Lowman and Somerville, 1976, Association JotAAH 2010). Lower scores reflect emaciation, and higher scores trend towards obesity, regardless of scale or species used for evaluation. For breeds of particular agricultural and economic importance–Holstein dairy cattle, for example–more detailed visual and textual scales may provide the best information for making management decisions balancing production efficiencies and animal health (Edmonson et al., 1989).

The Five Domains Model, the current international gold standard for conceptualizing sentient animal welfare, assesses an animal’s nutrition, physical environment, health, behavioral interactions, and mental state to describe its overall welfare status (Mellor et al., 2020). Varied tools to assess animal welfare employed by veterinarians, scientists, producers, public health officers, animal advocates, zookeepers, and conservationists across many domestic and wild species prominently feature BCS (Ghassani et al., 2023, Council VLCSA 2021, Health ISBoA 2025). This semi-quantitative data point can compare an individual to a healthy reference at a single point or track an animal’s body condition over time. Low and high BCS ratings can indicate an imbalance in an animal’s environment, physiology, or psychology. Across species, body condition is linked to livestock well-being and can be a useful indicator of welfare, disease status, hunger, and emotional state (Haskell, 2022, Harvey et al., 2023).

In veterinary practice and herd health management, BCS-based animal health assessment may be used to rapidly identify individuals for isolation, further examination, supplemental veterinary care, and diagnostic testing. Although subjective in nature, with appropriate training of personnel, the BCS assessment yields consistent and reliable results (Vieira et al., 2015). When integrated into comprehensive animal health assessments with other non-invasive measures of animal health, correlations between BCS and the incidence of infectious and parasitic diseases have been demonstrated in livestock and wildlife populations (Mansouryar et al., 2018, Sánchez et al., 2018). In addition to monitoring disease burden in a herd, leveraging BCS to manage livestock welfare appropriately can improve farmers’ production yields. In beef cattle, for example, there is a well-established relationship between BCS and reproductive health (Farney et al., 2024). As a tool implemented for comprehensive animal care, BCS has the potential to safeguard human health through a variety of One Health (OH) applications, underscoring the system’s utility as a valuable indicator for disease management, economic stability, and food security.

There is growing recognition of the value of measuring animal welfare in low- and middle-income countries (LMICs) globally with some tools beginning to gain traction for working equids (Sommerville et al., 2018). In Bangladesh, implementing routine welfare assessment including BCS on small dairy farms found correlations between low BCS and decreased milk production, and led to suggestions for improved husbandry practices (Islam et al., 2020). In Cambodia, BCS evaluation was used to assess beef production systems across distinct agro-ecological zones (Samkol et al., 2015). LMIC settings that require veterinary care for animals of economic and nutritional importance to be performed in low-resource environments are particularly strong candidates for implementing rapid, non-invasive assessment tools such as BCS. In the Democratic Republic of the Congo (DRC), the system of distributed smallholder farming is challenged by limited infrastructure, ongoing armed conflict, lack of access to veterinary care, and an observed but not routinely well-characterized burden of endemic livestock disease (Cishesa et al., 2022, Akilimali et al., 2023, Mugumaarhahama et al., 2021). The context of the DRC’s production livestock system sets the stage for BCS as a useful management tool for both assessing general herd welfare and as a potential early identifier of disease outbreaks. In cattle, strong relationships between BCS scores and susceptibility to disease (low, starvation state) or alternatively, metabolic disorders (high BCS) have been characterized (Loker et al., 2012).

To date, there have been no reports of semiquantitative BCS implemented in the DRC. Generally, assessments of animal nutrition, health, and welfare by farmers, researchers, and clinicians use qualifying adjectives (e.g., “cachectic”, “thin”, “good”, “fat”, “excellent”, “overweight” or “obese”) to describe fat reserves during different production phases. These unstandardized empirical evaluations, especially within the decentralized veterinary disease monitoring system of the DRC. This sporadically collected information is used for diagnostic and welfare evaluation to inform management, nutrition, and therapeutic approaches. However, uncoordinated and unstandardized empirical evaluation cannot report reliably report animal health status and can exacerbate issues presented by a decentralized national disease monitoring system in the DRC.

Here, we describe the introduction and validation of a context-specific, six-point semiquantitative, standardized scoring system for cattle and swine introduced in nine Congolese provinces. Given the limited standardization of animal health assessment tools, we also sought to evaluate inter-rater performance of this instrument at a subset of facilities across the DRC. We report initial findings and associations between livestock management practices, site characteristics, and individual BCS, recommending the continued integration of BCS into routine veterinary surveillance for general health and potential emerging zoonotic diseases in the DRC and other LMICs.

## Methods

A consortium of institutions based in the United States and DRC partnered to build and operationalize an inclusive model of ongoing veterinary surveillance for emerging infectious disease (EID) throughout the DRC *(publication under submission)*. Using a capacity-building collection methodology incorporating bidirectional information exchange, the investigation team gathered biological and epidemiological data from livestock, facilities, and humans working at these locations. The investigation team sought to strengthen existing local veterinary capabilities by using surveillance activities as educational opportunities for community animal health workers (CAHW), farm workers, farm owners, managers, and veterinarians at each site visited. Over 124 site visits to farms, abattoirs, and cooperative village herds were completed across nine provinces in the DRC between June 2023 and July 2024, the investigation team of Congolese veterinarians, public health partners, and veterinary technicians collected data spanning a variety of potential indicators for EID risk in bovine and porcine subjects. Data sources included serum specimens, attached ticks, and epidemiological information collected via tablet-based survey instruments. Beyond animal standard demographics, at the time of sampling, health indicators such as the presence of visible lesions, tick infestation, and records of recent illness as indicated by the facility manager were recorded. Indication of recent illness included any of the following clinical signs as noted by the facility manager: diarrhea, cough, wheezing or difficulty breathing, runny nose, discharge from eyes, lameness, fever, lethargy, inappetence, general malaise, colic, or pale mucous membranes. The BCS system presented subsequently was implemented at all sites to score bovine and porcine subjects included in the study. Individual bovine and porcine subjects were included if they were: found on the premises at the time of the investigation team’s site visit; able to be humanely restrained for blood collection from an accessible venipuncture site (coccygeal/tail or jugular vein); and had hydration and body mass sufficient to enable the safe collection of 6–8 mL of whole blood, as determined by the professional opinion of veterinarians and veterinary technicians staffing the investigation team.

### BCS as an interspecies scale

**Fig. 1:**
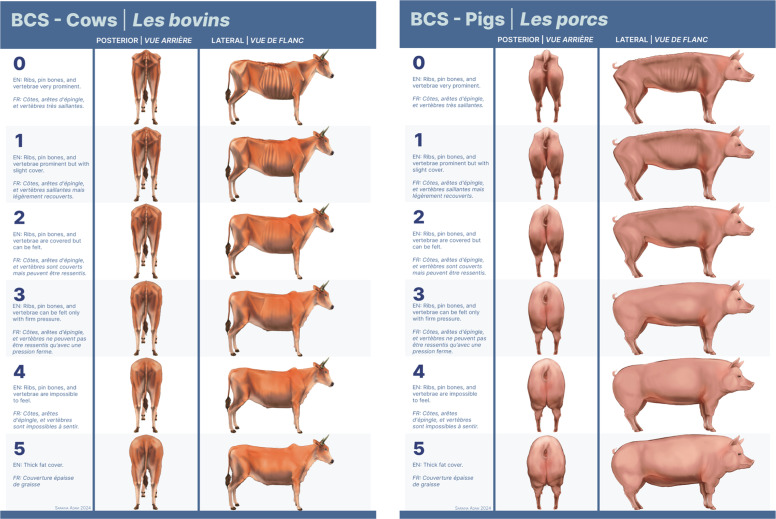
Visual reference materials with written descriptions of BCS for cattle (A) and swine (B).

This approach to BCS evaluation implemented in the DRC is adapted to traditional farming practices where multiple breeds and species may be mixed in the same herd or enclosure. Unlike many Western industrial agricultural settings that favor the intensive production of single species exclusively for meat or dairy, farmers in the DRC have livestock breeds selected for characteristics that improve tolerance of trypanosomes and tick-borne pathogens, survival in dry or hot conditions, and provide both meat and dairy as food and income sources (Mugumaarhahama et al., 2021). Given these constraints to utilizing a traditional single-species, single-breed BCS system, Congolese veterinary technicians staffing the investigation team were trained in a multi-species, harmonized BCS evaluation using a standardized six-point ordinal scale (zero to five) for the subjective assessment of cattle and swine muscle mass and fat deposition at key distributed points, as described in Table 1. While this scale spans the same six points for both species, specifics of fat deposition and anatomical differences are highlighted for each species (Fig. 1). Consistent assessment of all categories highlighted in Table 1 was intended to capture information essential to determining an accurate BCS for either porcine or bovine subjects, independent of their anatomical differences. In addition to descriptive text, team members were also provided visual reference materials (Coffey and Laurent, 1999, Katerji et al., 2004) during training. This scale was developed by referencing two baseline assessment systems–one bovine (Katerji et al., 2004) and one porcine (Soni et al., 2019)–to improve consistency and implementation of BCS evaluation while maintaining interspecies applications. Within the context of the scale’s application in the field, when a score of zero was assessed, the animal was alive but in a state of cachexia so severe that long-term survival was unlikely.

**Table 1:** BCS training descriptions for Porcine and Bovine Subjects.

Score	Visual Appearance	Pelvic Bones and Tail Head	Loin	Backbone	Ribs
0	Emaciated	Pelvic bones very prominent. Deep cavity around tail head.	Very narrow. Sharp edges on transverse spinal process. Flank very hollow.	Vertebrae prominent and sharp throughout length of backbone.	Individual ribs very prominent.
1	Poor	Pelvic bones obvious but some slight cover. Cover around tail head.	Loin narrow. Only slight cover to edge of transverse spinal process. Flank rather hollow.	Vertebrae prominent.	Rib cage is apparent but less prominent than above.
2	Moderate	Pelvic bones covered.	Edge of transverse spinal process covered and rounded.	Vertebrae visible over shoulder. Some cover further back.	Ribs covered but can be felt.
3	Ideal	Pelvic bones only felt with firm pressure. No cavity around tail.	Edge of transverse spinal process only felt with firm pressure. Flank full.	Vertebrae only felt with firm pressure.	Rib cage not visible. Very difficult to feel any ribs.
4	Overweight	Pelvic bones impossible to feel. Tail head set deep in surrounding fat.	Impossible to feel bones. Flank full and rounded.	Impossible to feel vertebrae.	Ribs impossible to feel.
5	Obese	Further deposition of fat impossible.	Further deposition of fat impossible.	Midline appears as slight hollow between rolls of fat.	Thick fat cover.

### BCS reference for Congo Basin bovine & swine species

To further improve the implementation of BCS assessment in the DRC, the investigation team integrated pictorial and experiential data from site visits spanning the DRC to develop BCS reference material spanning different species and subspecies of cattle and swine commonly found in the Congo Basin (Fig. 1). For bovines, this included indicine (*Bos indicus*) cattle (commonly known as “zebu” (Site TC 2024)), in particular Africander, Bonsmara, Ankole, Nelore, Mbororo, and Bukedi breeds, as well as taurine (*Bos taurus*) breeds such as N'dama, Friesland, and Swiss Brown (Sciences DoAaF 2024, Sciences DoAaF 2024). For swine (*Sus scrofa domesticus*), the Landrace, Large White, Large Black, Duroc, Piétrain breeds, and other local breeds were included (Briggs, 1969, Briggs, 1983, Sciences DoAaF 2024). For all species, breeds resulting from the crossbreeding of the above breeds were also considered. All reference illustrations were generated using Procreate and Adobe Illustrator 2024, with additional 3D modeling using Blender 3.5.

### BCS inter-rater reliability

To investigate agreement between raters and confirm the validity of the BCS assessment as implemented, in June 2024, the investigation team collected a series of paired ratings comparing outcomes scored by the standard palpation methodology to visual-only assessment. Demonstrating concordance between measures gathered by palpation compared to those by visual inspection furthers the ease of system use to incentivize livestock owners and managers to implement the system as a basic welfare assessment tool. To conduct the assessment, a veterinary staff member and trained technician both provided blinded BCS ratings of the same individual animal. The trained observer first completed a visual-only assessment, inspecting cranial-caudal, caudal-cranial, lateral, and dorsal-ventral views as permitted by restraint technique. This rating was logged prior to a second visual- and palpation-based rating by a second trained technician.

### Statistical analysis

All statistical analyses were completed using SAS 9.4 (SAS Institute Inc., Cary, NC, USA). Associations between binary health indicators and demographic variables with BCS ratings were evaluated using a Mann-Whitney-Wilcoxon test (Sundjaja et al., 2020). For categorical variables such as site type, species, province and age category with more than two independent groups, p-values were reported using the Kruskal-Wallis test (Bewick et al., 2004). Interrater reliability between the visually assessed dataset and the palpation-assessed dataset was determined using Cohen’s kappa score (κ). To detect a kappa score of 0.50 or greater between two raters at 80 percent power or better, a target of 25 samples was established (Sim and Wright, 2005). Positive kappa coefficient thresholds indicate agreement between raters as follows: 0.01–0.20 as none to slight, 0.21–0.40 as fair, 0.41– 0.60 as moderate, 0.61–0.80 as substantial, and 0.81– 1.00 as almost perfect agreement (McHugh, 2012).

### Ethical considerations

All activities conducted by the study team were in accordance with applicable institutional and national guidelines for the care and use of animals. This research was specifically approved by the University of California, Los Angeles Institutional Animal Care and Use Committee (IACUC, approval number ARC-2023-009). In the DRC, the study was approved by the Scientific Committee of the Faculty of Veterinary Medicine at the Université Pédagogique Nationale (Approval No. FMV/CS/023/2024) and by the Ethics Committee of the School of Public Health at the University of Kinshasa (Approval No. ESP/CE/46/2023).

## Results

Between June 2023 and July 2024, the investigation team deployed the BCS system in 124 site visits across nine provinces to score 1038 swine and 1157 bovine subjects–both beef (n = 1046) and dairy (n = 111) cattle (Table 2). Among all subjects, the investigation team noted 4.2 % with visible lesions, 2.2 % with recent illness, and 27.7 % with ticks present.

**Table 2:** Sample facility and animal characteristics.

	N	%
**Facility characteristic**	**n** = **124**	
Site Type		
Abbatoir	7	5.7
Farm	114	91.9
Village Herd	3	2.4
Province		
Equateur	16	12.9
Kasai	9	7.2
Kasai Oriental	10	8.1
Kinshasa	36	29.0
Lualaba	10	8.1
Maniema	10	8.1
Sud Ubangi	10	8.1
Tanganyika	12	9.7
Tshopo	11	8.9
**Animal characteristic**^1^	**n** = **2195**	
Species		
Dairy Cattle	111	5.1
Beef Cattle	1046	47.7
Swine	1038	47.3
Sex		
Male	748	34.1
Female	1447	66.0
Sterilization Status		
Neutered	178	8.1
Intact	2013	91.7
*Missing*	*4*	*0.2*
Age		
Infant (0 - 8 months)	152	6.9
Juvenile (8 - 15 months)	677	30.8
Adult (15 months - 10 years)	1345	61.3
Senior (10 - 20 years)	19	0.9
Geriatric (20 - 30 years)	2	0.1
BCS, all (median, interquartile range)	4	[3-4]
**Health Indicators At Sampling**		
Presence of Lesions	91	4.2
Indication of Illness	49	2.2
Presence of Ticks	607	27.7

1 Reported as n (%) unless otherwise noted.

**Fig. 2:**
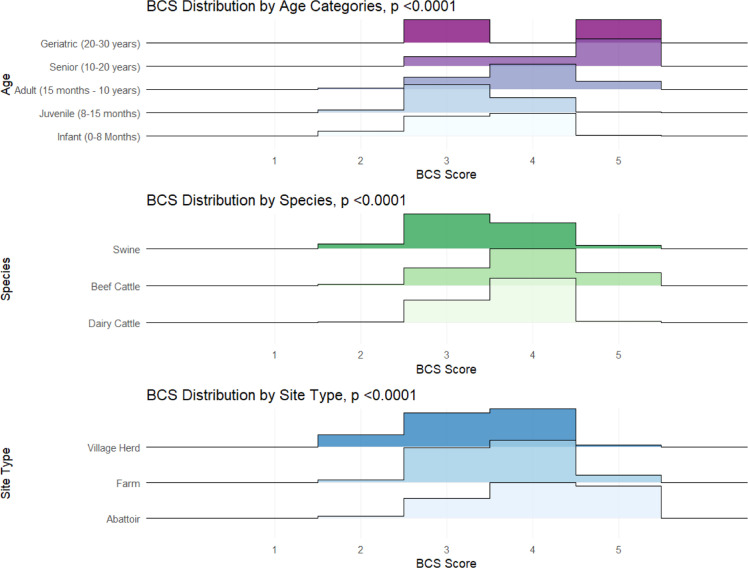
**BCS Distribution by Age, Species and Site Type.** P-values are reported as calculated by the Kruskal-Wallis test; the y-axis of a ridge plot designates the density of distribution.

Overall, the mean reported BCS across all species was 3.7, indicating good health across all sites sampled. By species, mean BCS were 3.7, 3.9, and 3.4 for dairy cattle, beef cattle, and swine, respectively. Significant variation in BCS ratings across species (p < 0.0001) and age category (p < 0.0001) of the sampled cattle and swine was also observed (Fig. 2, Table 3). For abattoirs, farms, and village herds sampled, Kruskal-Wallis tests revealed significant associations between BCS and the site-type of the animal sampled (p < 0.0001).

**Table 3:** Statistical measures of association for select binary and categorical variables.

Exposure	Chi-Square	DF	P-Value
**Wilcoxon**			
Reported Illness in the past 14 days			<0.0001
Lesions Identified			0.0899
Ticks Present			<0.0001
Sex			0.2937
Sterilization Status			0.0046
**Kruskal-Wallis**			
Age	286.4	4	<0.0001
Species	235.6	2	<0.0001
Site Type	75.8	2	<0.0001
Province	192.9	8	<0.0001

**Fig. 3:**
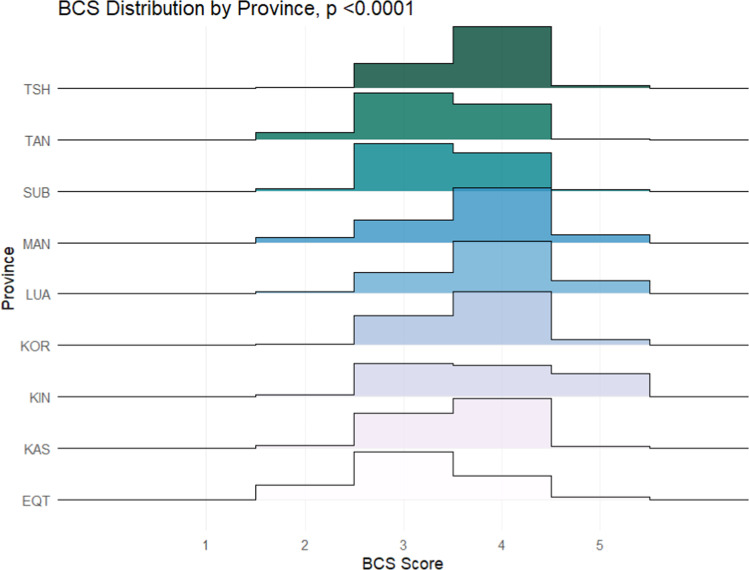
BCS Distribution by Province.

Beyond the multispecies utility of the BCS system, this scale was evaluated across provinces of different environmental and ecological regions of the DRC. Significant differences in median BCS were observed between species evaluated in the nine selected provinces of Equateur (EQT), Kasai (KAS), Kasai Oriental (KOR), Kinshasa (KIN), Lualaba (LUA), Maniema (MAN), Sud Ubangi (SUB), Tanganyika (TAN) and Tshopo (TSH) (Fig. 3, Table 3).

**Fig. 4:**
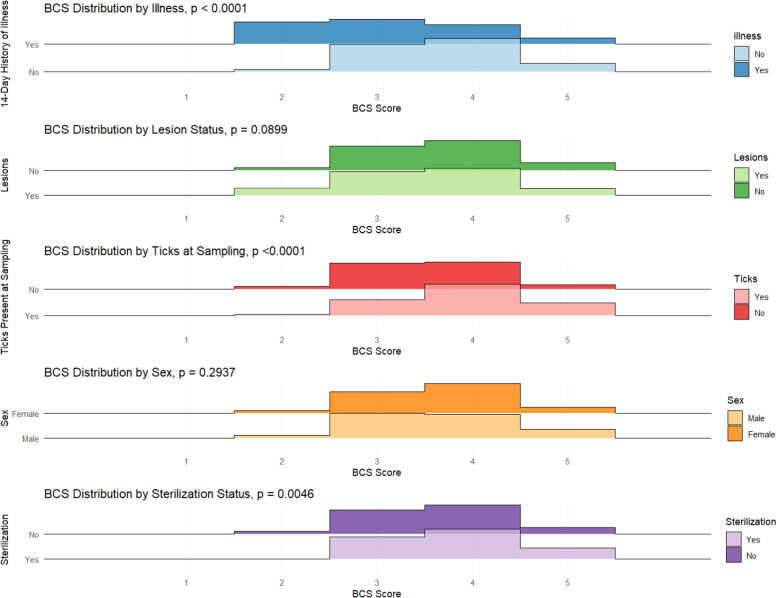
Wilcoxon Tests of Association between Selected Health Indicator Variables and BCS.

Health indicators at sampling such as a history of illness in the past 14 days, visible lesions and the presence of ticks were also noted by the sampling team. Wilcoxon tests indicated significant associations between illness (p < 0.0001), ticks (p < 0.0001), and sterilization status (p = 0.0046) and reciprocal BCS of individual animals. No significant association (p = 0.2937) between the sex of the animal or lesions at the time of sampling (p = 0.0899) and BCS were observed (Fig. 4, Table 3).

**Fig. 5:**
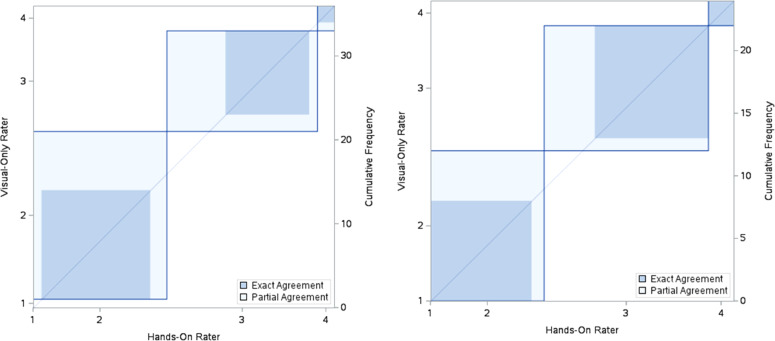
**(A)** Agreement between hands-on rater and visual-only rater for cattle and swine (n = 36). **(B)** Agreement between hands-on rater and visual-only rater for cattle (n = 26).

Following national implementation, across three sites in Kinshasa province, 36 individual animals were selected for assessment of inter-rater reliability of the BCS system. These included 10 swine and 26 cattle which were scored by 1 of 2 trained LVC technicians with visual and hands-on evaluation and 1 trained USDA team member with visual-only grading. Considering data points from swine and cattle together, it is unlikely that agreement is due to chance (κ = 0.4748, 95 % CI: 0.2206–0.7290) (Fig. 5**A**). In cattle, the BCS system was shown to be implemented with low likelihood that inter-rater agreement was due to chance alone (κ = 0.6407, 95 % CI: 0.3554–0.9260) (Fig. 5**B**). Agreement for swine only not due to chance was not proven by this data set (κ = 0.0769, 95 % CI: −0.3201–0.4740). Agreement in BCS scores in cattle samples were markedly improved in cattle (moderate agreement) compared to pigs (poor agreement). Scores were not spread further than 1 unit for each animal evaluated (n = 36).

## Discussion

Over the past several decades, BCS systems have been a mainstay of animal welfare and herd health management worldwide. This DRC-specific system is unique in its context-appropriate visuals and cross-species practicality. Traditionally, BCS systems have been tailored to a specific species or class of animal, for example, the nine-point BCS system for beef cattle (Lowman and Somerville, 1976). However, in an environment where beef cattle, dairy cattle, and swine (among other species) can be reared together in a village herd setting, a standardized six-point (0–5) scale provides a functional, efficient approach for veterinarians and other CAHW to assess herd health.

Here, we report use of a DRC-specific six-point scale for evaluating bovine and porcine health across nine geographically distinct provinces of the DRC. By age, species and site type, we show varied reported BCS scores across both included species—trends generally indicate increasing BCS scores with age, and higher average scores in cattle than pig subjects. Additionally, we demonstrate differences in mean BCS scores by province (p < 0.0001) indicating geographic differences in general semi-quantitative animal health.

In the case of animal diseases, except those diseases with a peracute or acute course, adiposity or emaciation are often considered a mirror of health disorders. As an indicator of herd health, standardized BCS indicating this adiposity or emaciation can trigger prevention or control efforts of a herd's suspected or ongoing disease (Roche et al., 2013). Notably, this study reports differences in BCS scoring by species and site type, with animals sampled at abattoirs with the higher mean BCS. This is aligned with the understanding that animals selected at abattoirs are more likely to have more adipose tissue, as their weight is increased before slaughter (Bruns et al., 2004). Further, this result provides evidence that healthier animals are brought for slaughter, ensuring a healthier meat supply. Importantly, the distribution of BCS was decreased among those animals with a reported history of illness and those with ticks at the time of sampling. These results further support the assertion that ratings using this cross-species BCS system can accurately reflect general changes in animal health due to reported illness or tick infestation.

By implementing a nested assessment of interrater reliability, this study also demonstrated the durability of a standard BCS system across two unique raters. Given the higher correlation (κ = 0.6407) between raters when assessing cattle specifically as opposed to pigs (κ = 0.0769), this may indicate that the BCS system was more appropriate or better understood for cattle assessments than their swine counterparts. The poor agreement in the porcine sub-sample may be attributed to difficulties assessing a visual-only BCS score in Congolese cross-bred swine, who have variations in integument thickness and hair texture as well as overall conformation that may be more consistently scored with a hands-on rating. However, this smaller nested assessment only included 36 animals total (across both cattle and pigs) and did not account for the veterinary background of the individual raters—one may be more familiar with cattle anatomy than the other, a potential driver of these differences in correlation. While further validation is merited, significant inter-rater agreement between cattle assessments does indicate the success of implementing this BCS system across breeds intended for both dairy and beef production, which is unique among other breed-specific BCS systems.

Anecdotally, investigation team technicians reported that the system was easy to understand and learn. In the field, team members discussed the value of BCS scoring with site owners, managers, and animal handlers, and trained these CAHW with the system itself. In the DRC, livestock practices and health surveillance are characterized by limited infrastructure, economic instability, limited capacity for monitoring animal diseases, and difficulties in accessing veterinary care and medicines. Yet, the implementation and cross-country training of CAHW with this BCS system–as well as knowledge transfer of proper restraint techniques–received strong support from local farmers, veterinarians, and CAHW participants of this study.

Among village farmers, the BCS system was seen as an innovation that allows for both a qualitative and quantitative assessment of the health status of their herds. At a higher provincial level, veterinarians and animal health practitioners supported the adoption of the BCS system, as these actors understood the managerial, health, and economic implications (assessment of market value) of a standardized system to improve animal welfare. Of note, in abattoirs and slaughterhouses, animal owners and butchers tended to directly correlate the assigned BCS with the commercial value of the animal by extrapolating from body fat status to animal welfare with higher scores indicating good herd management (e.g., disease prevention, proper veterinary care, and housing) and proper nutrition. The economic valuation of these animals by the BCS instrument, and interspecies nature of this rating can serve to professionalize the agriculture system both at a local and provincial level. Furthermore, studies have linked higher cattle BCS scores in American beef with profitability, further emphasizing the profitability aspect of BCS valuations (Scott et al., 2024). Integrating BCS into management and processing discussions may standardize language and empower producers to negotiate maximum market value for their livestock. Internationally, BCS can be used as an accepted measure of animal welfare and potential early indicator of disease outbreak. Implementing BCS to track trends in livestock wellbeing in the DRC may enable access to international funding and market opportunities with demonstrated, data-supported improvements in animal welfare.

This study is not without its limitations. First, animal inclusion criteria specifically excluded those animal subjects from which the study team could not successfully collect a blood specimen. By systematically excluding those animals without a body mass sufficient for safe blood collection, this introduces selection bias. Furthermore, the poor agreement in porcine scoring necessitates further investigation. While this agreement may be attributed to variance of morphological features of Congolese cross-bred swine that warrant palpation for most accurate BCS assessment rather than a visual-only rating, future investigations should seek to specify improved swine-specific landmarks such as intersection of the last rib with the loin and shoulder musculature. Additional training of local personnel would also serve to bolster the longevity of the BCS instrument as a functional tool used outside of the research context. Future investigations should focus on cross-training of all animal handlers—leading to improved provincial adoption of the BCS as both a health and economic metric.

While this specific BCS instrument was unique to the DRC context, evaluations of BCS systems are not. A recent standardized interspecies BCS system for tropical farm animals utilizes an identical six-point scale for large and small ruminants across the tropics globally (Vall et al., 2025). Here, this study creates a DRC-specific scale with references to those species specifically reared across the country. Additionally, work has been conducted to evaluate the use of a camera-assisted BCS evaluation of cattle (Zin et al., 2020). Given the low absolute error of this approach, future investigations could also incorporate those landmarks identified by the camera approach as key identifiers in better defining this instrument.

Ultimately, this study demonstrated the successful implementation of a semi-quantitative, cross-species six-point BCS system in the DRC. By creating a system specifically designed to reflect the species most commonly raised by commercial entities and village farmers across the country, this BCS scoring system was administered across cattle and swine in nine unique provinces. Differences in BCS distributions across these provinces indicate the versatility of the BCS system and its utility in comparing animal health across species, sites, and ecologically diverse environments for more holistic national agricultural assessments.

## Ethics in publishing statement

I testify on behalf of all co-authors that our article submitted followed ethical principles in publishing.

All authors agree that:

This research presents an accurate account of the work performed, all data presented are accurate and methodologies detailed enough to permit others to replicate the work.
This manuscript represents entirely original works and or if work and/or words of others have been used, that this has been appropriately cited or quoted and permission has been obtained where necessary.
This material has not been published in whole or in part elsewhere.
The manuscript is not currently being considered for publication in another journal.
That generative AI and AI-assisted technologies have not been utilized in the writing process or if used, disclosed in the manuscript the use of AI and AI-assisted technologies and a statement will appear in the published work.
That generative AI and AI-assisted technologies have not been used to create or alter images unless specifically used as part of the research design where such use must be described in a reproducible manner in the methods section.
All authors have been personally and actively involved in substantive work leading to the manuscript and will hold themselves jointly and individually responsible for its content.

## CRediT authorship contribution statement

**Paige Rudin Kinzie:** Writing – review & editing, Writing – original draft, Methodology, Investigation, Formal analysis. **Sydney Merritt:** Writing – review & editing, Writing – original draft, Visualization, Methodology, Investigation, Formal analysis, Data curation. **Boniface Lombe:** Writing – review & editing, Project administration, Methodology, Investigation. **Megan Halbrook:** Writing – review & editing, Supervision, Project administration, Investigation, Conceptualization. **Danae Witte:** Writing – review & editing, Methodology, Investigation. **Emmanuel Hasivirwe Vakaniaki:** Writing – review & editing, Project administration, Methodology, Investigation. **Saraina Adam:** Writing – review & editing, Visualization, Software, Methodology. **Alex Madidi L’k’ye:** Writing – review & editing, Investigation, Data curation. **Patrick K. Mukadi:** Writing – review & editing, Supervision, Project administration, Investigation. **Milette Mfwankang:** Writing – review & editing, Investigation. **Jean Mona:** Writing – review & editing, Investigation. **Daddy Kanonge:** Writing – review & editing, Investigation. **Carleigh Melofchik:** Writing – review & editing, Resources, Methodology, Investigation. **Jean-Jacques Muyembe-Tamfum:** Writing – review & editing, Supervision, Resources. **Nicole A. Hoff:** Writing – review & editing, Supervision, Project administration, Investigation, Funding acquisition, Conceptualization. **Lisa E. Hensley:** Writing – review & editing, Supervision, Resources, Funding acquisition. **Placide Mbala-Kingebeni:** Writing – review & editing, Supervision, Resources, Conceptualization. **Augustin Twabela:** Writing – review & editing, Supervision, Resources, Project administration, Conceptualization. **Anne W. Rimoin:** Writing – review & editing, Supervision, Resources, Funding acquisition, Conceptualization.

## Declaration of competing interest

The authors declare that they have no known competing financial interests or personal relationships that could have appeared to influence the work reported in this paper.

## References

[bib0018] Akilimali A., Biamba C., Fasawe A.S., Cakwira H., Oduoye M.O., Ojo-Akosile T. (2023). One Health” outlook in the Democratic Republic of Congo. IJS Global Health.

[bib0002] Association JotAAH (2010).

[bib0031] Bewick V., Cheek L., Ball J. (2004). Statistics review 10: further nonparametric methods. Critical Care.

[bib0027] Briggs HM. (1969).

[bib0028] Briggs HM. International Pig Breed Encyclopedia: Elanco Animal Health; 1983.

[bib0035] Bruns K., Pritchard R., Boggs D. (2004). The relationships among body weight, body composition, and intramuscular fat content in steers. Journal of Animal Science.

[bib0017] Cishesa T.H., Kavange A.B., Kulimushi J.Z., Rugendabanga D.C.O., K’Eka C.N.K., Rosine R.L. (2022). Traditional livestock system and pathologies risk in the small-scale pig farming in Kamituga sub county, Eastern Democratic Republic of the Congo. Open Access Library Journal.

[bib0021] Coffey RD PG, Laurent K.M. (1999).

[bib0006] Council VLCSA. Use of body condition scoring in animal welfare investigations. In: vermont agency of agriculture FaM, editor.; 2021. p. 2.

[bib0003] Edmonson A., Lean I.J., Weaver L., Farver T., Webster G. (1989). A body condition scoring chart for Holstein dairy cows. Journal of Dairy Science.

[bib0013] Farney JK BD, Johnson S., Reinhardt C., Tarpoff A.J., Waggoner J., Weaber R. (2024).

[bib0005] Ghassani Y.K., Rianti P., Priambada N.P., Arifin I., Saptorini I., Prameswari W. (2023). Welfare assessment of slow loris (Nycticebus spp.) at an Indonesian primate rehabilitation center: Development and validation of body condition score. American Journal of Primatology.

[bib0009] Harvey A.M., Beausoleil N.J., Ramp D., Mellor DJ. (2023). Mental experiences in wild animals: scientifically validating measurable welfare indicators in free-roaming horses. Animals.

[bib0008] Haskell M. (2022).

[bib0007] Health ISBoA. Animal care & welfare resource materials. Current information, hot topics 2025 [cited 2025; Available from: https://www.in.gov/boah/current-information/hot-topics/animal-care-and-welfare-resource-materials/#Livestock.

[bib0015] Islam M.A., Sharma A., Ahsan S., Mazumdar S., Rudra K., Phillips CJ. (2020). Welfare assessment of dairy cows in small farms in Bangladesh. Animals.

[bib0022] Katerji M., Mougin E., d'Herbès J.M., Hiernaux P., Kergoat L. (2004).

[bib0020] Loker S., Miglior F., Koeck A., Neuenschwander T.F.O., Bastin C., Jamrozik J. (2012). Relationship between body condition score and health traits in first-lactation Canadian Holsteins. Journal of Dairy Science.

[bib0001] Lowman BG SN, Somerville SH. Condition scoring of cattle. East Scotland college of agriculture. 1976;Bull. 6.

[bib0011] Mansouryar M., Mirzaei-Alamouti H., Banadaky M.D., Nielsen M. (2018). Calving body condition score combined with milk test data and rectal tempreture improved the prognostic value of non-invasive markers for infectious diseases in Holestein cows. Livestock Science.

[bib0033] McHugh ML. (2012). Interrater reliability: the kappa statistic. Biochemia Medica (Zagreb).

[bib0004] Mellor D.J., Beausoleil N.J., Littlewood K.E., McLean A.N., McGreevy P.D., Jones B. (2020). The 2020 five domains model: including human-animal interactions in assessments of animal welfare. Animals (Basel).

[bib0019] Mugumaarhahama Y., Ayagirwe R.B.B., Mutwedu V.B., Cirezi N.C., Wasso D.S., Azine P.C. (2021). Characterization of smallholder cattle production systems in South-Kivu province, eastern Democratic Republic of Congo. Pastoralism.

[bib0034] Roche J.R., Kay J.K., Friggens N.C., Loor J.J., Berry DP. (2013). Assessing and managing body condition score for the prevention of metabolic disease in dairy cows. Veterinary Clinics of North America: Food Animal Practice.

[bib0016] Samkol P., Sath K., Patel M., Windsor P.A., Holtenius K. (2015). Survey of smallholder beef cattle production systems in different agro-ecological zones of Cambodia. Tropical Animal Health and Production.

[bib0025] Sciences DoAaF. Breeds of Cattle: Africander Cattle. Breeds of Livestock 2024 [cited 2025; Available from: https://breeds.okstate.edu/cattle/africander-cattle.html.

[bib0026] Sciences DoAaF. Breeds of Cattle: N'dama Cattle. Breeds of Livestock 2024 [cited 2025; Available from: https://breeds.okstate.edu/cattle/ndama-cattle.html.

[bib0029] Sciences DoAaF. Breeds of Swine. Breeds of Livestock 2024 [cited 2025; Available from: https://breeds.okstate.edu/swine/.

[bib0036] Scott K.G., Wang Y., Bohrer B.M., Garcia LG. (2024). The effect of live body condition score of beef cows on carcass characteristics, carcass-cutting yields, processor profitability, and tenderness in the longissimus lumborum and psoas major muscles. Translational Animal Science.

[bib0032] Sim J., Wright CC. (2005). The kappa statistic in reliability studies: use, interpretation, and sample size requirements. Physical Therapy.

[bib0024] Site TC. Zebu. 2024 [cited 2025; Available from: https://www.thecattlesite.com/breeds/beef/76/zebu/.

[bib0014] Sommerville R., Brown A.F., Upjohn M. (2018). A standardised equine-based welfare assessment tool used for six years in low and middle income countries. Public Library of Science ONE.

[bib0023] Soni A., Mishra S., Singh N., Bobade M.D., Dubey A., Yadav A., Bhagat S., Verma U. (2019). Body condition scoring of swine: a review. International Journal of Chemical Studies.

[bib0030] Sundjaja JH, Shrestha R, Krishan K. McNemar And Mann-Whitney U Tests. 2020. 32809534

[bib0012] Sánchez C.A., Becker D.J., Teitelbaum C.S., Barriga P., Brown L.M., Majewska A.A. (2018). On the relationship between body condition and parasite infection in wildlife: a review and meta-analysis. Ecology Letters.

[bib0037] Vall E., Blanchard M., Sib O., Cormary B., González-García E. (2025). Standardized body condition scoring system for tropical farm animals (large ruminants, small ruminants, and equines). Tropical Animal Health and Production.

[bib0010] Vieira A., Brandão S., Monteiro A., Ajuda I., Stilwell G. (2015). Development and validation of a visual body condition scoring system for dairy goats with picture-based training. Journal of Dairy Science.

[bib0038] Zin T.T., Seint P.T., Tin P., Horii Y., Kobayashi I. (2020). Body condition score estimation based on regression analysis using a 3D camera. Sensors (Basel).

